# Role of Threat and Coping Appraisal in Protection Motivation for Adoption of Preventive Behavior During COVID-19 Pandemic

**DOI:** 10.3389/fpubh.2021.678566

**Published:** 2021-07-05

**Authors:** Arista Lahiri, Sweety Suman Jha, Arup Chakraborty, Madhumita Dobe, Abhijit Dey

**Affiliations:** ^1^Department of Community Medicine, College of Medicine and Sagore Dutta Hospital, Kolkata, India; ^2^COVID Patient Admission Cell, Swasthya Bhawan, Kolkata, India; ^3^Department of Preventive and Social Medicine, All India Institute of Hygiene and Public Health, Kolkata, India; ^4^Department of Community Medicine, Medical College and Hospital, Kolkata, India; ^5^Department of Health Promotion and Education, All India Institute of Hygiene and Public Health, Kolkata, India; ^6^World Health Organization RNTCP Technical Support Network, Swasthya Bhawan, Kolkata, India

**Keywords:** behavior, COVID-19, prevention, protection motivation theory, response efficacy, threat appraisal, self-efficacy

## Abstract

With more than 100 million cases and over 2 million deaths globally, the COVID-19 pandemic continues to remain a major threat. Identifying the behavioral factors influencing preventive behaviors for COVID-19 are crucial in devising public health policies to promote essential strategies to combat the pandemic in an efficient manner. The current study was therefore conducted to estimate the prevalence of COVID-19 preventive behaviors and measure their association with behavioral constructs like threat perception, response efficacy, and self-efficacy, as per socio-demographic background. A region-stratified online survey focusing on the constructs of protection motivation theory, for example, threat and coping appraisal for preventive health practices against COVID-19, was carried out among adult users of social media in India. Generalized linear models with cluster-adjusted-robust standard errors were used to analyze the responses and model the preventive practices among the study population. Analysis of a total 2,646 responses revealed that proper perceptions regarding cause, symptoms, and transmission of COVID-19 were prevalent in the majority of the respondents. The majority of the participants reported frequent use of face masks (93.20%), followed by frequent washing of hands with soap and water (84.90%). The majority of the respondents affirmed that, though not frequently but sometimes, they avoid touching the face with unclean hands. Frequently covering mouth with the crook of the elbow while sneezing and coughing, and maintaining physical distance when outside was noted among 74.14 and 83.84%, respectively. The proportion of participants frequently using sanitizers to clean hands and those infrequently practicing the same were comparable. Self-efficacy for preventive practices and threat-appraisal of COVID-19 illness were identified as important determinants of the selected COVID-19 preventive behaviors, independently. The analysis confirmed that practices of the behaviors were mostly synergistic to each other. Current findings highlight that formulation of precise risk communication strategies to improve perceptions regarding threat appraisal and self-efficacy could facilitate desirable practices, which are also effective in the prevention of airborne infections and, hence, may contribute toward broader policy directions. The evidence urges the implementation of precision-driven risk communication and diffusion of these practices to attain behavioral herd immunity.

## Introduction

The world is reeling under the ever-increasing threat of the novel coronavirus infection (COVID-19). There has been over 160 million cases of COVID-19 infection and more than 3 million deaths worldwide; however, in the second most populous country of the world, India, the corresponding figures are more than 27 million and 3 lakhs, respectively (1). In India, as the pandemic is wreaking havoc, fear is still lurking in the minds of the people. Theoretical models have already shown the impact of strict hygiene and quarantine measures in halting the epidemic (2–5). In such infectious disease pandemics, the willingness and compliance of the general public to recommendations regarding personal hygiene, or movement restrictions, may neither be self-evident nor self-motivated (6–8) but may depend largely on the fear appeals and stressful situation. In order to understand how people behave and cope during stressful situations, the protection motivation theory (PMT) was put forward, emphasizing the intrinsic and extrinsic factors that can lead to motivation and performance of the desired behavior (9). This understanding is expected to be helpful in formulating precisely tailored persuasive communication.

**Figure 1:**
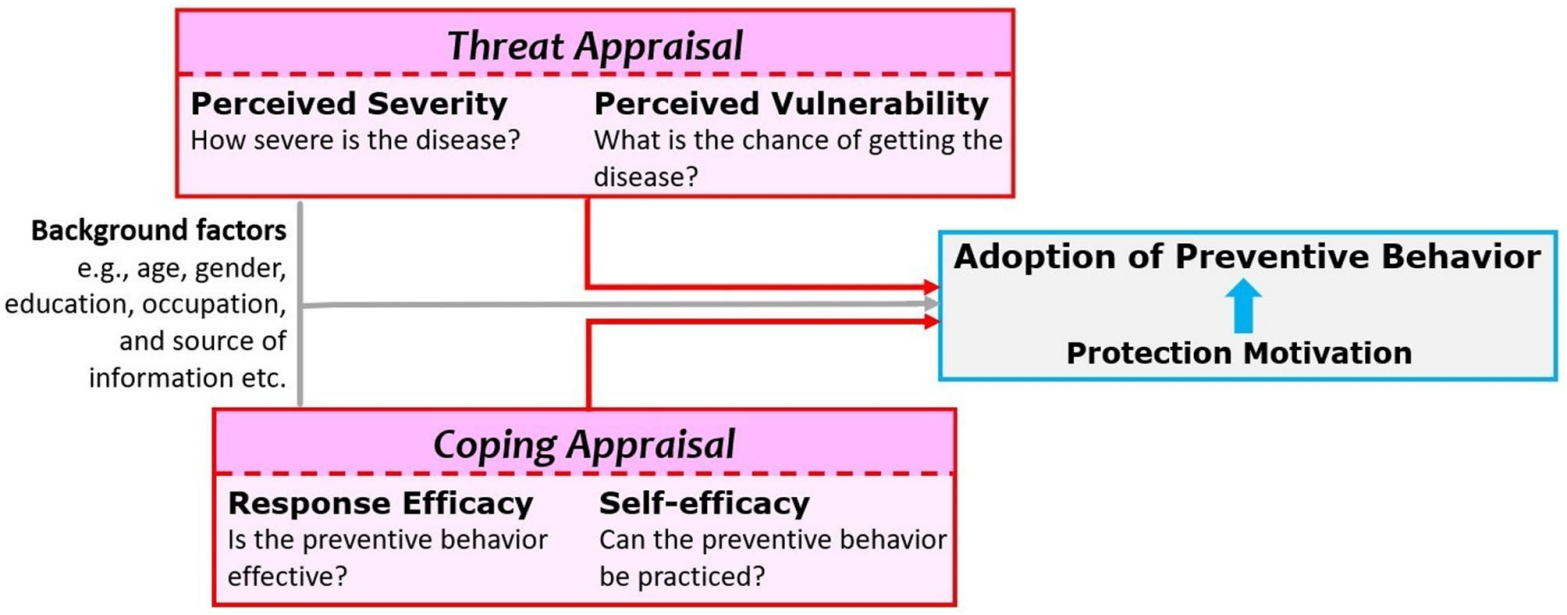
Protection motivation theory (PMT) framework adopted for the current study. Adoption (or practice) of preventive or protective behavior is immediately preceded by motivation for the same. According to PMT, it is a resultant of threat appraisal (perceived severity and perceived vulnerability) and coping appraisal (response efficacy and self-efficacy) adjusted for the background characteristics of the respondents.

The PMT framework involves threat appraisal and coping appraisal as the multidimensional determinants of motivation. Threat appraisal is the combination of perceived severity (perceptions regarding the degree of harm) and perceived vulnerability (perception regarding the chance that one will experience harm) regarding the situation, excluding the perceived rewards (positive aspects) of the situation. Coping appraisal experienced is the combination of response efficacy (belief in the effectiveness of the recommended behavior in removing or preventing possible harm) and self-efficacy (the belief that one can successfully enact the recommended behavior), subtracting the response costs (the perceived or actualized costs associated with practice of the recommended behavior). These constructs intrinsic to the model ultimately lead to a protection motivation to perform adaptive responses (in this case recommended COVID-preventive behaviors). However, building on the experiences gathered during previous major outbreaks like severe acute respiratory syndrome (SARS) and hemagglutinin type 1 and neuraminidase type 1 Influenza (H1N1 Influenza), the threat appraisal of COVID-19 in terms of perceived vulnerability and perceived severity along with coping appraisal of protective behaviors in terms of response efficacy and self-efficacy were presumed to be the major determinants of preventive practices (10–15). It has also been conceptually proposed in the current study that the practice of one particular behavior is influenced by the practice of other preventive behaviors. The recommended preventive behaviors may also be affected by factors beyond these constructs, e.g., age, gender, occupation, education, knowledge, and personal experiences (14, 16–23). Social media also have immense motivational value (24, 25). A working framework utilized in the current study has been presented in Figure 1.

In order to understand the dynamics of these factors in practicing preventive behaviors, six key behaviors, that is, handwashing with soap and water, using hand sanitizers when soap was not available, using a face mask, avoiding touching face without cleaning hands, using the crook of the elbow to cover mouth and nose while sneezing and coughing, and maintaining a physical distance of at least 2 m when outside, were selected (26, 27). Currently, no studies are known to have measured the preventive practices or analyzed the determinants of these practices among the Indian population. The current study was based on the PMT to explain why people engage in unhealthy practices and offers suggestions for changing those behaviors through precise risk communication strategies. Risk communication and related perceptions as a basis for the desired behavioral change have not been studied adequately, especially in the Indian context despite several studies predicting the trajectory of the outbreak in the light of different preventive strategies (4, 28, 29). The aim of the current research was thus to measure the association between practice of the selected preventive behaviors and threat perception, response efficacy, and self-efficacy, adjusting for socio-demographic background.

## Materials and Methods

### Study Design and Participants

An analytical online questionnaire-based survey was conducted among social media users from India. The data collection for this study was conducted in a single wave from May 16 to August 2, 2020. Individuals, who had access to social media platforms like Facebook^®^ and/or Twitter^®^ and/or Instagram^®^ and/or LinkedIn^®^, were considered as the study population. Adult population (18–65 years) and Indian by nationality who were currently living in India since the beginning of the nation-wide lockdown on March 25, 2020 were included in the study. Those who reported to have a critical illness or receiving palliative care or who reported having suffered COVID-19 prior to the study were excluded. Participants diagnosed with any cognitive or psychiatric illness or those who reported being on psychotropic or sedative medication were also excluded from this study through skip patterns incorporated in the online questionnaire.

### Selection of the Participants

An online pilot study focusing on the selected preventive measures was performed among 74 active users of social media platforms residing in states of eastern and northern India, before the start of the current survey. An overall proportion of ~35% for using the crook of the elbow to cover mouth and nose while sneezing and coughing was the lowest practiced preventive behavior. Considering this proportion with 5% precision and 90% power of the study, applying a design effect of 2 and a nonresponse proportion of 40%, the sample size was calculated to be 2,282. In order to calculate a corrected minimum sample size, a correction factor for “successful spread of questionnaire” was introduced. “Successful spread of questionnaire” was defined as the number of completed responses obtained (through social media spread or shares) after a primary participant disseminated (shared) the questionnaire. Now, considering this “successful spread of questionnaire proportion” to be 0.1, the corrected minimum sample size was 2,074. Taking the six zones in India as sampling strata, the target sample size in each stratum was ~346.

**Figure 2:**
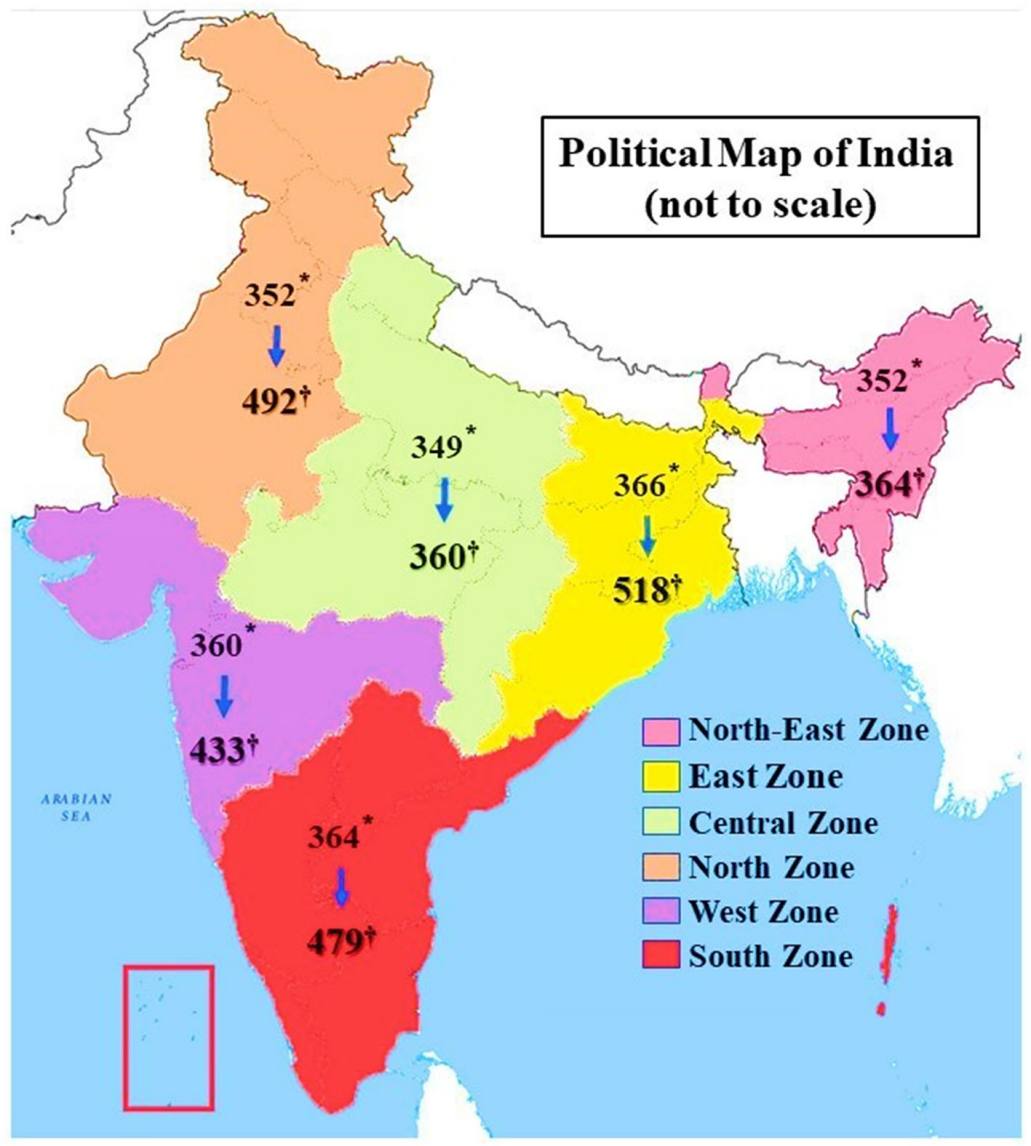
Zones in India and participants selected from each zone. *The number of participants in the respective zone who were primarily given the data collection form (represents only the primary respondents), ^†^ Who completed and submitted the form (includes primary respondents and also those who responded through spread of the questionnaire). Both these numbers represent eligible study population only after applying inclusion and exclusion criteria.

The zonal construction and the states within are shown in Figure 2 and Supplementary Table 1 (refer Supplementary File 1). The names of these states were used as inclusive search terms to identify participants based on their residence (as registered in their profiles). The resultant open-ended consecutively extracted and cleaned list was used as a sampling frame, and the desired number of participants in different zones were selected through random sequences. The participants were contacted through their available contact information (email or WhatsApp^®^ number) and the Google form^®^ was shared. Finally, a total of 2,646 responses were included in the final analysis with 518 from the Eastern zone, 492 from the Northern zone, 433 from the Western zone, 479 from the Southern zone, 360 from the Central zone, and 364 from the Northeastern zone. The details of questionnaire distribution and response rates are provided in Supplementary Table 2 (refer Supplementary File 1).

### Measurements

#### Study Tool

The questionnaire was developed with the help of a brainstorming session with five subject experts from the disciplines of epidemiology, psychology, psychiatry, and health promotion. The preliminary questionnaire was pre-tested on a group of 30 adults with variable educational and occupational backgrounds. The final online questionnaire had sections on demographic details (age, sex, residence, occupation, and education), knowledge about COVID-19 (common symptoms and modes of transmission), and primary source of information about preventive behaviors, the self-reported practice of preventive behaviors, threat appraisal (perceived vulnerability and perceived severity), and coping appraisal (response efficacy and self-efficacy). Awareness about common symptoms and modes of transmission were a multiple-response set of questions. Questions on practice and appraisal constructs were on a 3-point Likert-type scale. The reliability of the questionnaire was assessed by domain-specific discrimination and difficulty parameters using an item response model (30, 31) and was found to be satisfactory.

#### Preventive Practices

The respondents were enquired about their usual frequency of practice of the six selected health behaviors, for example, handwashing with soap and water, using hand sanitizers when soap was not available, using a face mask, avoiding touching face without cleaning hands, using the crook of the elbow to cover mouth and nose while sneezing and coughing, and maintaining a physical distance of at least 2 m when outside, each graded into frequent/regular, sometimes, and rare.

#### Threat Appraisal

The perceived threat was identified through three questions, that is, vulnerability to COVID-19 with progressing time (temporal vulnerability), vulnerability compared to other people (interindividual vulnerability), and vulnerability due to the area of residence (spatial vulnerability). Perceived severity was explored through the generalized perception of the disease severity. Each response was graded high, same (neither high nor low), and low.

#### Coping Appraisal

Response efficacy measuring the perception of participants about the effectiveness of each preventive behavior was recorded in a three-point Likert-type scale ranging from “very effective” to “not effective at all.” For self-efficacy, the confidence to practice each preventive behavior was measured in another three-point scale ranging from “very confident” to “not confident at all.” Response efficacy and self-efficacy questions were included in separate sets for each of the preventive practices in this study. For example, in the case of “using face mask” as a preventive behavior, respective response efficacy and self-efficacy questions were placed together along with the self-reported frequency of practice and similarly for other behaviors as well.

### Statistical Analysis

#### Primary Analysis

Statistical analysis was performed using the STATA 14.0 software (StataCorp LLC, College Station, TX, USA). Confidentiality was maintained while cleaning and storing the data for analysis. The responses to demographic and knowledge questions were used to understand the background of respondents. Prevalence of different categories of self-reported practice frequency for the selected preventive behaviors was calculated with robust standard errors and adjusted for clustering, also weighing for the region (strata)-specific response rate. The practice frequency questions were dichotomized. Category of infrequent practice combined “sometimes” and “rarely” responses, and the other category was frequent practice. In order to understand the effects of different predictors, adjusted prevalence ratio (aPR) with 95% CI was calculated through the Poisson regression models with robust cluster-adjusted standard errors built separately to predict “frequent practice” of each of the selected behaviors (32, 33). For each model, along with threat appraisal variables and coping appraisal (for that particular preventive practice being modeled) variables, frequency and demographic variables of other practices were included as predictors. Threat appraisal and coping appraisal questions were also dichotomized for the regression models. The highest perceptions of each threat appraisal item were contrasted against combining the other response categories (e.g., “same” and “low”). In the case of response efficacy (and self-efficacy) questions, “very effective” (or “very confident”) category was contrasted against combining “somewhat” and “not at all” categories. For statistical inferencing, *p* < 0.05 was taken as significant.

#### Handling of Missing Data

Missing data were handled through exclusion from the analysis. For reporting the prevalence of an item, completed responses for that particular item were included for calculation. However, when performing inferential statistics, only responses that were complete for all the variables included in that analysis were analyzed. Similarly, in case of the six independent regression models, the total number of responses analyzed varied. This was because only those responses having non-missing data points for all the variables included in a particular regression model were utilized.

### Ethics

Clearance was obtained from the Institutional Ethics Committee (MC/KOL/IEC/NON-SPON/730/07/2020). Those who participated in the study provided online informed consent before responding to the online questionnaire. No incentives were provided for responding and/or dissemination of the questionnaire.

## Results

### Socio-Demographic Information

The socio-demographic profile of the participants is depicted in Table 1. Among the respondents, the majority were male (62.28%), aged ≤ 35 years (43.08%), were currently married (65.76%), were residing in urban areas (86.36%), and were professional degree holders (61.25%). Among those currently employed, 33.95% were regularly attending workplaces.

**Table 1:** Socio-demographic profile of the participants.

**Socio-demographic profile**	***N* (%)**
**AGE GROUPS (*****n*** **=** **2,646)**	
≤ 35 years	1,140 (43.08)
36–50 years	602 (22.75)
≥51 years	904 (34.17)
**GENDER (*****n=*** **2,646)**	
Male	1,648 (62.28)
Female	998 (37.72)
**RESIDENCE (*****n=*** **2,646)**	
Urban	2,285 (86.36)
Rural	361 (13.64)
**MARITAL STATUS (*****n=*** **2,646)**	
Currently married	1,740 (65.76)
Not currently married	906 (34.24)
**EDUCATIONAL STATUS (*****n=*** **2,640)**	
Up to completed higher secondary	121 (4.58)
Graduates and above (not professionals)	902 (34.17)
Graduates and above (professionals)	1,617 (61.25)
OCCUPATIONAL STATUS^*^ (***n=*** 2,646)	
Currently employed	1,682 (63.57)
Currently studying	481 (18.18)
Going to workplace/institute	674 (25.47)
Currently healthcare worker	679 (25.66)
CURRENTLY LIVING WITH^*^ (***n*** = 2,372^†^)	
Spouse	1,557 (65.64)
Parents and/or parents in-law	1,250 (52.70)
Grandparents and/or grandparents in-law	100 (4.22)
Children and/or son-in-law/daughter-in-law	1,180 (49.75)
Friends and/or other people	183 (7.72)

*“n” represents the number of completed responses for respective variables. “N (%)” represents the number (percentage) corresponding to the categories*.

* *Multiple response*.

† *Those who are not living alone*.

### Awareness Related to COVID-19

Fever, cough, and sore throat were identified as symptoms of COVID-19 infection by more than 90% of respondents. Droplets and person-to-person transmission were reported as the key routes of spread by 94.75 and 91.46% of participants. The participants primarily obtained information about preventive practices from news media (45.38%) and health personnel (32.11%). Awareness-related data are given in Table 2.

**Table 2:** Awareness about symptoms and transmission of COVID-19 and information about preventive practices.

**Awareness about COVID-19**	***N* (%)**
**SYMPTOMS OF COVID-19 (*****n*** **=** 2,646)^*^	
Fever	2,592 (97.96)
Cough	2,566 (96.98)
Sore throat	2,448 (92.52)
Running nose	1,576 (59.56)
Body-ache	1,976 (74.68)
Fatigue/tiredness	2,067 (78.12)
Other symptoms	1,769 (66.86)
**ROUTES OF TRANSMISSION OF COVID-19 (*****n*** **=** 2,646)^*^	
Person to person	2,420 (91.46)
Animal to person	455 (17.20)
Via droplets	2,507 (94.75)
Via faeco-oral route	1,222 (46.18)
*Other*	920 (34.77)
**PRIMARY SOURCE OF INFORMATION ON COVID-APPROPRIATE PREVENTIVE BEHAVIORS (*****n*** **=** **2,638)**	
Informed by health personnel	847 (32.11)
Social media	440 (16.68)
News media	1,197 (45.38)
Informed by non-healthcare worker	154 (5.83)

“*n” represents the number of completed responses for respective variables. “N (%)” represents the number (percentage) corresponding to the categories*.

* *Multiple response*.

### Practice of COVID-Appropriate Behaviors

Table 3 depicts the prevalence of self-reported preventive practices. Frequent washing of hands with soap and water was reported by 84.90% (95% CI: 83.59–86.12%). Frequently using a mask, covering mouth with the crook of the elbow while sneezing and coughing, and maintaining physical distance when outside were reported by 93.20% (95% CI: 92.45–93.86%), 74.14% (95% CI: 72.71–75.52%), and 83.84% (95% CI: 82.48–85.10%) participants, respectively. While the frequent practice of four (33.88%) or five (27.83%) preventive measures was common, all six measures were frequently practiced by 8.21%.

**Table 3:** Practice of different preventive behavior as reported by the participants.

**Practice of preventive behaviors**	**Number**	**Proportion (95% CI)**
**WASHING HANDS WITH SOAP AND WATER (*****n*** **=** **2,630)**		
Frequently	2,233	84.90 (83.59–86.12)
Sometimes	390	14.83 (13.66–16.07)
Rarely	7	0.27 (0.13–0.52)
**AVOID TOUCHING FACE WITH UNCLEAN HANDS (*****n=*** **2,630)**		
Frequently	976	37.11 (36.18–38.04)
Sometimes	1,258	47.83 (45.82–49.84)
Rarely	396	15.06 (13.37–16.90)
**REGULARLY CLEANING HANDS WITH SANITIZER (*****n*** **=** **2,631)**		
Frequently	1,252	47.57 (44.70–50.44)
Sometimes	1,090	41.44 (38.82–44.11)
Rarely	289	10.98 (10.05–11.99)
**COVER MOUTH WITH THE CROOK OF THE ELBOW WHILE SNEEZING AND COUGHING (*****n*** **=** **2,631)**		
Always	1,951	74.14(72.71–75.52)
Sometimes	548	20.84 (19.29–22.46)
Rarely	132	5.02 (4.57–5.50)
**MASK TO COVER NOSE AND MOUTH (*****n*** **=** **2,631)**		
Always	2,452	93.20 (92.45–93.86)
Sometimes	153	5.82 (51.69–65.40)
Rarely	26	0.99 (0.90–1.08)
**MAINTAINING DISTANCE WHEN OUTSIDE (*****n*** **=** **2,631)**		
Always	2,206	83.84 (82.48–85.10)
Sometimes	387	14.71 (13.49–16.02)
Rarely	38	1.44 (1.13–1.83)

*All the Confidence Intervals (CIs) were calculated with the help of robust standard error estimation technique accounting for clustering for sampling zones. “n” represents the number of completed responses for respective variables*.

### Threat Appraisal and Coping Appraisal

Threat appraisal in terms of perceived vulnerability and perceived severity of COVID-19 illness is shown in Table 4. Among those who perceived the disease to be highly severe, the majority considered themselves to be highly vulnerable to COVID-19. Perception of higher vulnerability with the temporal progression of the pandemic was noted in 58.24% of those who perceived the severity of the disease to be like other common illnesses. Higher levels of perceived vulnerability were found among those who had a higher perceived severity, which was statistically significant.

**Table 4:** Threat appraisal related to COVID-19.

**Perceived vulnerability to COVID-19**	**Perceived severity of the disease**			***p*-value**
	**Lower (*n =* 678)**	**Same (*n =* 1,025)**	**Higher (*n =* 925)**	
**With progression of pandemic (time)**				
Lower (*n =* 361)	161 (23.75)	108 (10.54)	92 (9.95)	0.000
Same (*n =* 743)	168 (24.78)	320 (31.22)	255 (27.57)	
Higher (*n =* 1,524)	349 (51.47)	597 (58.24)	578 (62.49)	
**In comparison with others**				
Lower (*n =* 746)	360 (53.10)	203 (19.80)	183 (19.78)	0.000
Same (*n =* 1,084)	200 (29.50)	616 (60.10)	268 (28.97)	
Higher (*n =* 798)	118 (17.40)	206 (20.10)	474 (51.24)	
**Due to current residence area**				
Lower (*n =* 1,100)	362 (53.39)	404 (39.41)	334 (36.11)	0.000
Same (*n =* 704)	146 (21.53)	335 (32.68)	223 (24.11)	
Higher (*n =* 824)	170 (25.07)	286 (27.90)	368 (39.78)	

*Figures within first bracket indicate column-percentage of the row-categories. “n” signifies number of responses analyzed in the mentioned categories, considering only the completed responses in the pairs of variables. The p-values were calculated by χ^2^ test for the statistical association*.

Self-efficacy and response efficacy about COVID-appropriate preventive behaviors are depicted in Table 5. The association of response efficacy and self-efficacy for each of the selected COVID-appropriate behaviors was observed to be statistically significant. Better response efficacy was associated with a better self-efficacy.

**Table 5:** Coping appraisal of different preventive behaviors.

**Variables**	**Response efficacy**	**Self-efficacy**				***P*-value**
		**Very much**	**Somewhat**	**Not at all**	**Total**	
**Regular handwash with soap and water**	Very much	2,099 (89.05)	251 (10.65)	7 (0.30)	2,357 (100.00)	0.000
	Somewhat	132 (54.55)	110 (45.45)	0 (0.00)	242 (100.00)	
	Not at all	20 (74.07)	0 (0.00)	7 (25.93)	27 (100.00)	
	Total	2,251 (85.72)	361 (13.75)	14 (0.53)	2,626 (100.00)	
**Avoid touching face with unclean hands**	Very much	1,483 (63.40)	765 (32.71)	91 (3.89)	2,339 (100.00)	0.000
	Somewhat	76 (31.54)	136 (56.43)	29 (12.03)	241 (100.00)	
	Not at all	22 (46.81)	7 (14.89)	18 (38.30)	47 (100.00)	
	Total	1,581 (60.18)	908 (34.56)	138 (5.25)	2,627 (100.00)	
**Frequently using sanitizer to clean hands**	Very much	1,644 (76.47)	472 (21.95)	34 (1.58)	2,150 (100.00)	0.000
	Somewhat	148 (33.71)	239 (54.44)	52 (11.85)	439 (100.00)	
	Not at all	20 (52.63)	7 (18.42)	11 (28.95)	38 (100.00)	
	Total	1,812 (68.98)	718 (27.33)	97 (3.69)	2,627 (100.00)	
**Covering mouth and nose with crook of elbow**	Very much	1,855 (86.72)	272 (12.72)	12 (0.56)	2,139 (100.00)	0.000
**while sneezing and coughing**	Somewhat	146 (32.59)	254 (56.70)	48 (10.71)	448 (100.00)	
	Not at all	24 (61.54)	0 (0.00)	15 (38.46)	39 (100.00)	
	Total	2,025 (77.11)	526 (20.03)	75 (2.86)	2,626 (100.00)	
**Using mask to cover mouth & nose**	Very much	2,197 (93.93)	127 (5.43)	15 (0.64)	2,339 (100.00)	0.000
	Somewhat	127 (52.05)	107 (43.85)	10 (4.10)	244 (100.00)	
	Not at all	24 (54.55)	1 (2.27)	19 (43.18)	44 (100.00)	
	Total	2,348 (89.38)	235 (8.95)	44 (1.67)	2,627 (100.00)	
**Maintaining distance at least 2 m with others**	Very much	1,913 (83.72)	327 (14.31)	45 (1.97)	2,285 (100.00)	0.000
	Somewhat	136 (45.48)	133 (44.48)	30 (10.03)	299 (100.00)	
	Not at all	25 (58.14)	11 (25.58)	7 (16.28)	43 (100.00)	
	Total	2,074 (78.95)	471 (17.93)	82 (3.12)	2,627 (100.00)	

*Figures within parentheses represent percentages. The p-values are calculated by the Chi-square test with continuity correction. Only the completed responses for the pairs of variables were considered for the Chi-square test between respective pairs*.

### Factors Associated With COVID-19 Preventive Behaviors

Table 6 summarizes the factors associated with each preventive practice. Being informed by any healthcare worker about the COVID-appropriate behaviors was more effective in facilitating the adoption of preventive practices among respondents. The frequency of a preventive practice was not statistically associated with the perceived efficacy of the practice, except for regular cleaning of hands with sanitizers. Practice of the preventive behaviors had statistically significant association with their respective self-efficacy. The effects (aPR) of self-efficacy were considerably more than response efficacy in this regard.

**Table 6:** Prevalence ratios (95% CI) of the predictors of self-reported practices of selected COVID-appropriate preventive behaviors.

	***Model A:***		***Model B:***		***Model C:***		***Model D:***		***Model E:***		***Model F:***	
	**Frequently washing hands with soap and water**		**Frequently avoid touching face without cleaning hands**		**Frequently cleaning hands with sanitizer**		**Frequently covering mouth with crook of elbow while sneezing and coughing**		**Regular use of mask to cover nose and mouth**		**Regularly maintaining appropriate physical distance when outside**	
	**(*****n*** **=** **2,615)**		**(*****n*** **=** **2,617)**		**(*****n*** **=** **2,617)**		**(*****n*** **=** **2,616)**		**(*****n*** **=** **2,617)**		**(*****n*** **=** **2,617)**	
	**aPR (95% CI)**	***p*-value**	**aPR (95% CI)**	***p*-value**	**aPR (95% CI)**	***p*-value**	**aPR (95% CI)**	***p*-value**	**aPR (95% CI)**	***p*-value**	**aPR (95% CI)**	***p*-value**
***Higher*** **vulnerability of the participants to COVID-19, with progression of pandemic (time)** (Ref.: Lower or same)	1.01 (1.00–1.02)	0.009	1.10 (0.99–1.22)	0.060	0.92 (0.91–0.94)	0.000	0.99 (0.95–1.04)	0.757	1.01 (0.99–1.02)	0.147	0.93 (0.91–0.95)	0.000
***Higher*** **vulnerability of a respondent to COVID-19 in comparison to others** (Ref.: Lower)	1.03 (1.01–1.05)	0.014	0.87 (0.60–1.26)	0.452	1.21 (1.14–1.28)	0.000	1.02 (0.98–1.07)	0.265	0.94 (0.92–0.95)	0.000	1.01 (0.99–1.03)	0.266
***Higher*** **vulnerability of the participants to COVID-19, due to current residence area** (Ref.: Lower)	1.0.00 (0.97–1.02)	0.934	1.70 (1.37–2.11)	0.000	1.13 (1.10–1.16)	0.000	0.99 (0.98–1.02)	0.939	1.06 (1.05–1.07)	0.000	0.98 (0.97–0.98)	0.000
***Higher*** **perceived severity of the disease compared to existing reports** (Ref.: Lower)	1.01 (0.98–1.04)	0.523	0.97 (0.77–1.23)	0.818	0.91 (0.86–0.97)	0.003	0.93 (0.89–0.97)	0.000	1.06 (1.03–1.09)	0.000	0.99 (0.97–1.02)	0.560
**This particular preventive behavior is very effective in prevention of COVID-19** (Ref.: Somewhat or not effective)	0.98 (0.93–1.04)	0.565	1.37 (0.82–2.30)	0.228	1.23 (1.14–1.32)	0.000	0.99 (0.89–1.11)	0.919	0.97 (0.93–1.02)	0.297	0.99 (0.94–1.03)	0.526
**Very confident to practice this particular preventive behavior** (Ref.: Somewhat or not confident)	1.66 (1.53–1.80)	0.000	1.01 (0.94–1.09)	0.744	2.87 (2.49–3.31)	0.000	2.52 (2.19–2.91)	0.000	1.35 (1.27–1.45)	0.000	1.78 (1.71–1.84)	0.000
**Frequently washing hands with soap and water** (Ref.: Sometimes or rarely)	*(Variable of interest in this model)*		0.74 (0.68–0.81)	0.000	2.19 (1.89–2.53)	0.000	1.05 (0.99–1.10)	0.063	1.01 (0.99–1.03)	0.134	1.11 (1.09–1.12)	0.000
**Frequently avoid touching face without cleaning hands** (Ref.: Sometimes or rarely)	0.97 (0.95–0.98)	0.000	(Variable of interest in this model)		1.31 (1.27–1.35)	0.000	1.03 (0.99–1.08)	0.114	0.95 (0.93– 0.98)	0.000	1.00 (0.99–1.02)	0.599
**Frequently cleaning hands with sanitizer** (Ref.: Sometimes or rarely)	1.19 (1.15–1.23)	0.000	2.02 (1.85–2.21)	0.000	(Variable of interest in this model)		1.05 (1.02–1.08)	0.001	1.04 (1.02–1.06)	0.000	0.98 (0.97–0.99)	0.041
**Frequently covering mouth with crook of elbow while sneezing and coughing** (Ref.: Sometimes or rarely)	1.12 (1.08–1.17)	0.000	1.44 (1.18–1.75)	0.001	1.12 (1.06–1.20)	0.000	(Variable of interest in this model)		1.13 (1.11–1.14)	0.000	1.06 (1.03–1.09)	0.000
**Regularly using mask to cover nose and mouth** (Ref.: Sometimes or rarely)	1.16 (1.08–1.24)	0.000	0.57 (0.42–0.77)	0.000	1.73 (1.48–2.02)	0.000	1.79 (1.66–1.93)	0.000	(Variable of interest in this model)		1.18 (1.12–1.25)	0.000
**Regularly maintaining appropriate physical distance when outside** (Ref.: Sometimes or rarely)	1.12 (1.10–1.15)	0.000	1.05 (0.96–1.15)	0.249	0.92 (0.83–1.01)	0.081	1.11 (1.08–1.15)	0.000	1.07 (1.05–1.09)	0.000	(Variable of interest in this model)	
**Age group** (Ref.: 18–35 Years)												
36–50 years	1.02 (0.97–1.06)	0.503	0.91 (0.76–1.08)	0.271	1.12 (1.05–1.20)	0.000	0.99 (0.95–1.02)	0.388	1.03 (1.00–1.07)	0.034	0.92 (0.89–0.94)	0.000
51–65 years	1.06 (1.04–1.09)	0.000	0.82 (0.59–1.15)	0.246	0.91 (0.83–0.99)	0.028	0.93 (0.91–0.94)	0.000	1.00 (0.99–1.02)	0.579	0.98 (0.96–1.01)	0.113
**Gender** (Ref.: Male)												
Female	1.00 (0.96–1.04)	0.914	0.98 (0.79–1.22)	0.888	0.94 (0.86–1.03)	0.167	0.99 (0.98–1.02)	0.983	0.99 (0.98–1.01)	0.143	1.07 (1.03–1.11)	0.000
**Residence and living arrangement**												
Rural (Ref.: Urban)	1.03 (0.99–1.06)	0.086	1.44 (1.21–1.71)	0.000	0.88 (0.83–0.95)	0.001	1.05 (1.01–1.09)	0.022	0.96 (0.94–0.99)	0.004	0.95 (0.92–0.99)	0.015
Living alone (Ref.: living with others)	1.03 (0.99–1.07)	0.201	0.78 (0.57–1.08)	0.136	0.96 (0.85–1.09)	0.578	1.04 (0.99–1.08)	0.074	1.02 (0.99–1.05)	0.238	0.94 (0.90–0.97)	0.001
**Educational qualification** (Ref.: Up to completed higher secondary level)												
Graduates and above with nonprofessional degrees	1.03 (0.96–1.10)	0.386	1.42 (0.94–2.16)	0.100	0.74 (0.69–0.79)	0.000	1.09 (1.03–1.15)	0.004	1.04 (0.99–1.09)	0.079	0.91 (0.86–0.96)	0.001
Professional degree (graduate and above)	1.01 (0.93–1.10)	0.763	1.82 (1.30–2.53)	0.000	0.71 (0.61–0.81)	0.000	1.08 (0.99–1.16)	0.065	1.03 (0.98–1.09)	0.221	0.92 (0.88–0.97)	0.002
**Occupational status**												
Going to workplace/institution on a regular basis (Ref.: not going on a regular basis)	1.01 (0.98–1.05)	0.444	0.64 (0.53–0.78)	0.000	1.05 (0.99–1.11)	0.075	0.97 (0.92–1.02)	0.226	1.02 (0.99–1.04)	0.168	1.03 (0.98–1.08)	0.216
Healthcare worker (Ref.: other than healthcare worker)	0.98 (0.96–0.99)	0.005	0.86 (0.69– 1.07)	0.179	1.01 (0.97–1.06)	0.639	0.99 (0.94–1.06)	0.995	1.00 (0.98–1.03)	0.714	0.96 (0.99–1.02)	0.164
**Primary source of information on preventive practices** (Ref.: Informed by a person other than healthcare worker)												
Informed by health personnel	1.10 (1.07–1.14)	0.000	0.91 (0.67–1.24)	0.559	1.09 (0.95–1.25)	0.208	1.16 (1.03–1.30)	0.017	0.98 (0.95–1.02)	0.326	1.12 (1.05–1.18)	0.000
Social media	1.06 (1.03–1.11)	0.001	0.89 (0.67–1.19)	0.454	1.44 (1.01–1.29)	0.028	1.13 (0.97–1.31)	0.131	0.99 (0.96–1.03)	0.750	1.14 (1.05–1.24)	0.002
News media	1.02 (0.98–1.06)	0.286	0.87 (0.68–1.13)	0.306	1.03 (0.92–1.15)	0.656	1.06 (0.97–1.16)	0.184	1.01 (0.96–1.06)	0.651	1.12 (1.05–1.20)	0.000

*aPR, adjusted prevalence ratio; Ref., Reference category. “n” represents the number of completed responses for all variables in the respective models. Log pseudo-likelihood for models, A: −2530.38, B: −1079.14, C: −1967.67, D: −2385.39, E: −2592.76, F: −2516.57; Akike's information criteria (AIC) for models, A: 1.94, B: 0.83, C: 1.50, D: 1.83, E: 1.98, F: 1.93; Bayesian information criteria (BIC) for model, A: −19921.98, B: −19185.59, C: 19112.53, D: −19652.23, E: −20246.35, F: −19908.74*.

Perception of higher vulnerability to COVID-19 with the progression of time was associated with frequent handwashing with soap and water, but infrequently cleaning hands with sanitizers, and occasionally maintaining a physical distance. Those who perceived vulnerability to the infection to be higher than other individuals reported increased prevalence of washing hands with soap and water and regular use of sanitizers to clean hands but a decreased prevalence of mask use. A higher perception of vulnerability owing to the place of residence of an individual was associated with better practices of avoiding touching face with unclean hands, cleaning hands with sanitizers, and using a mask when outside. Higher perceived severity was associated with frequent use of mask, infrequently using sanitizers, and infrequently covering mouth while sneezing and coughing. Practicing one of the behaviors frequently was observed to be associated with a better practice of other preventive behaviors with occasional exceptions.

## Discussion

### Key Findings

Majority respondents were aware of fever, cough, and sore throat as symptoms of COVID-19 disease. Major routes of spread considered were *via* droplet and person-to-person transmission. Regular use of face masks was the commonest preventive behavior. However, frequent use of hand sanitizers and avoidance of touching the face without cleaning the hands were less practiced. Regarding threat appraisal of COVID-19, those having a higher perception of vulnerability had higher perceived severity about the disease. Regarding coping appraisal, higher response efficacy was associated with higher self-efficacy for particular preventive behaviors. While coping and threat appraisals were associated with the practice of some of the preventive behaviors, self-efficacy was identified as the most important determinant of practicing COVID-appropriate behaviors. The practice of one preventive behavior was often associated with the practice of the other.

### What Is Already Known and What This Study Adds

Concurrent literature reported an acceptable level of awareness about COVID-19 illness (22, 34, 35). An Ethiopian study reported that around 95% of participants knew about droplet-mediated spread (35). The proportion was slightly higher than that observed in the current study. Min et al. documented that <10% of respondents correctly completed knowledge-related questions (8).

In general, researchers identified self-efficacy for practicing a preventive behavior to be the most important construct in the context of COVID-appropriate behaviors (19, 21, 22, 35–40). The current study findings support this notion. Researchers have rarely examined the role of response efficacy in preventive practices (19, 36, 37, 39, 41, 42). Sometimes, the response cost has been used in place of response efficacy to understand coping appraisal (21). The current study examined the role of response efficacy for all COVID-appropriate behaviors studied, but found statistical association only with frequently cleaning hands with sanitizers. However, in an online survey conducted in Iran, researchers found response efficacy to be overall significantly associated with intention to perform a behavior (40).

In this study, majority of the participants perceived that with time, vulnerability to COVID-19 will increase. The findings were in consonance with a study from China conducted during the early phases of H1N1 pandemic (43). The current study respondents also perceived that vulnerability to COVID-19 was lower because of the area of their residence—a finding that highlights the focal burden of the disease. Those who perceived higher vulnerability due to their area of residence practiced preventive behaviors frequently, except physical distancing. The underlying factor may be related to daily supply related issues and lockdown rules. Overall higher perceived severity and vulnerability were associated with better practice of some of the COVID-appropriate behaviors. Similarly, researchers have demonstrated indirect or direct effect of threat perception leading to better preventive practice (21, 22, 37, 39, 41, 42, 44, 45). With higher level of perceived severity, the practice of use of sanitizers, and covering mouth and nose with crook of elbow was lower. Probably, the latter was perceived as a difficult adaptive behavior. Those who apprehended an increased vulnerability in future, were found to have less practice of physical distancing and using sanitizers. Less frequent use of sanitizers in both the situations can be attributed to its overall low prevalence probably due to lack of availability. Higher perceived susceptibility compared to other individuals had poorer practice of mask use, but good prevalence of use of soap and water and sanitizers to clean hands. The lack of practice adoption may probably be an outcome of complex interplay in the risk-resilience framework (46). However, a study among Chinese nationals noted that negative or fear-linked emotions after controlling for trust factors led to poorer preventive behaviors, which were in stark contrast to most of the current findings (8).

Frequent practice of all the preventive measures were reported by <10% of the respondents, whereas >90% of the respondents confirmed that they were frequently practicing at least one of the preventive behaviors. Niu et al. reported a slightly lower prevalence of practicing at least one preventive behavior, but overall nearly half of the respondents were practicing all the preventive behaviors regularly (38). Use of face mask—the dominant preventive practice in the current study—was evidently higher than the reported evidence in another South Korean study (41).

In the current study, all the preventive practices were invariant of whether the respondent stayed with family or not, except physical distancing. This was in partial agreement with findings from a study conducted in North Carolina (22). Healthcare workers reported poorer preventive practice, for example, lower prevalence of frequent use of soap and water. This was in stark contrast to the findings reported from other countries, which may be due to the difference in selection of study population (37, 39, 45). The current study showed that some practices were significantly lower among the older population, which was not the case with other study findings, may be because of different social dynamics and poor focus on health of elderly (27, 35, 37). Gender was not related to practices, except maintaining physical distance when outside. However, researchers have mostly agreed on the fact that women perform preventive practices better than men (27, 34, 37). Those who received information primarily from social media were more prone to practice good preventive practices. The findings support the inference drawn by Chesser et al. in their study regarding public health activism in social media (25).

Authors have demonstrated simultaneous practice of several preventive behaviors in different populations and also in times of previous outbreaks, but evidence is lacking to demonstrate how the practice of one behavior is associated with the better practice of the other behavior (11–13, 21, 27, 34, 35, 37, 38, 41). This scope has been ushered by the proposed concept that similar behaviors will aid in the better practice of behavior. The findings from the regression models showed the following pairs of practices that were synergistic in nature: (1) use of soap water to clean hands and use of sanitizers, (2) use of soap water to clean hands and physical distancing, (3) use of sanitizers to clean hands and avoiding touching face without cleaning hands, (4) use of sanitizers and covering mouth and nose with a bent elbow while coughing and sneezing, (5) use of sanitizers and use of a mask to cover nose and mouth, (6) use of a mask and covering mouth and nose with a bent elbow, (7) covering mouth and nose with a bent elbow and physical distancing when outside, and (8) physical distancing and use of a mask. On the other hand, some pairs of practices were found to be inversely related: (1) use of soap and water to clean hands and avoiding touching face without cleaning hands and (2) avoiding touching face without cleaning hands and use of face mask. Interestingly, a higher frequency of the use of sanitizer was associated with a poorer practice of physical distancing, though physical distancing did not statistically predict sanitizer use.

Supporting the hypothesis, the practice of similar behaviors was found to be independent predictors of another behavior, along with the constructs of the PMT framework. The behaviors were separately influenced by individual efficacy constructs, but threat perceptions remained the same for all. It may therefore be argued from a statistical perspective that the predictor behaviors were exogenous in each of the models because the models were built independently of each other.

### Strengths and Limitations

Though some studies have utilized the PMT framework, the current study is the first one to utilize the framework for demonstrating the effects of different behavioral constructs in the practice of COVID-appropriate behaviors in India. Also, the behavioral constructs like threats and coping appraisals have been adjusted for the effect of practicing similar behaviors. Additionally, response efficacy, which lacked due importance while testing behavior frameworks, has been addressed appropriately in the current study. The present study also provides insights into the role of self-efficacy in the practice of COVID-appropriate behaviors. The current study utilized an open-frame sampling technique to address the sampling-related challenges in an online survey. The precision of the results, despite variability in response proportions of several predictors, is founded on the use of generalized linear models for the statistical analysis with robust estimation methods. Still, the results can only be generalized to those who have had regular access to social media during the period of data collection because of the survey design adapted. The scenario should therefore be considered as the tip of the iceberg.

Self-reported responses are often considered biased, but response validation with retest among a random sample was helpful for the data integrity and validity. The variability in the proportion of practice may have been diluted with distinction bias (47). While some behaviors may have been difficult to adapt, cognitive compensation might have resulted in a framing effect in the participants' responses to these questions (48). Although the relationship of different practices is an important finding along with the effects of threat perception and efficacy constructs, the opposing effects noted may be interpreted in light of these probable biases.

## Conclusions

The concept of synergistic practices can be theoretically incorporated in the PMT model for predicting the likelihood of adopting precautionary behavior. The present study identified that only few participants were practicing all preventive behaviors frequently. With restrictions eased off in the midst of a considerable case burden, only rigorous practice of COVID-19 preventive behaviors along with effective vaccination can help contain further propagation of infection. Although media campaigns proved effective in making people adopt some of these behaviors, the focus should now be on promoting synergistic behavioral practices through risk communication. The results of the current study are limited to the users of social media, but if they can be provided with adequate awareness and motivation, then the desirable practices are expected to achieve diffusion among a larger section of the people. It was found that news media was the major source of information about preventive practices followed by healthcare workers warranting focused campaigns through these sources. The evidence urges formulation of strategies for risk communication for behavior change in a targeted manner, ensuring diffusion of the preventive practices for attaining behavioral herd immunity against airborne infections in the long term.

## Data Availability Statement

The original contributions presented in the study are included in the article/Supplementary Material, further inquiries can be directed to the corresponding author/s.

## Ethics Statement

The studies involving human participants were reviewed and approved by Institutional Ethics Committee, Medical College and Hospital, Kolkata. The participants were provided with an online informed consent document. Only those who agreed to this online consent, responded and participated in the study.

## Author Contributions

AL and SJ: conceptualization, methodology, software, formal analysis, investigation, writing—original draft, and project administration. AC: methodology, validation, formal analysis, and writing—review and editing. MD: conceptualization, methodology, formal analysis, writing—review and editing, and supervision. AD: software, formal analysis, and writing—review and editing. All authors contributed to the article and approved the submitted version.

## Conflict of Interest

The authors declare that the research was conducted in the absence of any commercial or financial relationships that could be construed as a potential conflict of interest.

## Supplementary Material

Supplementary File 1 Constitution of sample strata and selection of participants.
