# Supplementary material for: Role of Threat and Coping Appraisal in Protection Motivation for Adoption of Preventive Behavior During COVID-19 Pandemic
Source: Front Public Health. 2021 Jul 5;9:678566. doi: 10.3389/fpubh.2021.678566 (PMC8287502; doi:10.3389/fpubh.2021.678566)
Supplement: Supplementary File 1 — Constitution of sample strata and selection of participants. [file Table_1.pdf]

Selection of participants according to composition of zonal strata:

Table S1.1. Composition of the zonal strata in terms of different states of India.

Zone (Sampling Strata)	States
**East Zone**	Bihar, Jharkhand, Odisha, and West Bengal
**North Zone**	Chandigarh, Delhi, Haryana, Himachal Pradesh, Jammu and Kashmir, Ladakh, Punjab, and Rajasthan
**West Zone**	Dadra and Nagar Haveli and Daman and Diu, Goa, Gujarat, and Maharashtra
**South Zone**	Andhra Pradesh, Karnataka, Kerala, Puducherry, Tamil Nadu, Telangana, Andaman and Nicobar Islands, Lakshadweep.
**Central Zone**	Chhattisgarh, Madhya Pradesh, Uttarakhand and Uttar Pradesh
**North-East Zone**	Sikkim, Assam, Arunachal Pradesh, Manipur, Meghalaya, Mizoram, Nagaland and Tripura

Zone-wise distribution of response numbers:

Table S1.2. Zone-wise distribution of response dynamics among the participants.

Strata	Primary distribution [a]	Added (Secondary) distribution [b]	Number of participants approached based on sampling protocol [c]	Number of participants responded among originally approached (based on sampling zone) [d]	Response rate [e]	Responses obtained as per zonal migration (based on current residence) [f]	Migration proportion among respondents [g]	Total number of respondents including responses from questionnaire spread by the participants (based on current residence)
**East zone**	354	89	443	370	0.84	391	0.06	518
**North Zone**	340	91	431	369	0.86	389	0.05	492
**West Zone**	305	55	360	292	0.81	245	- 0.16	433
**South Zone**	276	88	364	269	0.74	294	0.09	479
**Central Zone**	311	38	349	266	0.76	274	0.03	360
**North-East Zone**	296	79	375	281	0.75	254	- 0.10	364

[a] Participants who were provided with the google form based on sampling strategy.

[b] Further distribution was done to a second set of participants when response from the primary participant was not obtained within one week of questionnaire delivery.

[c] Number of participants approached based on sampling protocol = {Primary distribution + Added (Secondary) distribution}

[d] Shows the number of participants who responded, based on their zone as per their digital profile (i.e. as per the sampling strategy)

[e] Response rate was calculated as, {Number of participants responded (based on sampling zone) ÷ Number of participants approached based on sampling protocol}

[f] Number of participants classified based on their current zone of residence. This number varies from the 'Number of participants responded (based on sampling zone)', because the digital profiles were not updated about their current residence. There were participants who migrated from the sampling zone to their current residence' zone.

[g] Migration proportion among respondents = $\frac{\{\text{Responses obtained as per zonal migration (based on current residence)} - \text{Number of participants responded (based on sampling zone)}\}}{\text{Number of participants responded (based on sampling zone)}}$
